# Acceptability of condom promotion and distribution among 10–19 year-old adolescents in Mpwapwa and Mbeya rural districts, Tanzania

**DOI:** 10.1186/1471-2458-12-569

**Published:** 2012-07-29

**Authors:** Amon Exavery, Godfrey M Mubyazi, Jovitha Rugemalila, Adiel K Mushi, Julius J Massaga, Hamisi M Malebo, Filemon Tenu, Joyce K Ikingura, Sia Malekia, Emmanuel A Makundi, Acleus SM Ruta, John W Ogondiek, Victor Wiketye, Mwelecele N Malecela

**Affiliations:** 1 Ifakara Health Institute, P.O. Box 78373, Dar es Salaam, Tanzania; 2 National Institute for Medical Research (NIMR), Headquarters, P.O. Box 9653, Dar es Salaam, Tanzania; 3 National Bureau of Statistics (NBS), P.O. Box 796, Dar es Salaam, Tanzania; 4 Amani Medical Research Centre, P.O Box 81, Muheza, Tanzania; 5 Tanga Medical Research Centre, P.O Box 950, Tanga, Tanzania; 6 Ngongongare Medical Research Centre, P.O Box 514, Usa River, Arusha, Tanzania

**Keywords:** Acceptability, Condom promotion and distribution, Adolescents, Tanzania

## Abstract

**Background:**

The HIV/AIDS pandemic remains a leading challenge for global health. Although condoms are acknowledged for their key role on preventing HIV transmission, low and inappropriate use of condoms persists in Tanzania and elsewhere in Africa. This study assesses factors affecting acceptability of condom promotion and distribution among adolescents in Mpwapwa and Mbeya rural districts of Tanzania.

**Methods:**

Data were collected in 2011 as part of a larger cross-sectional survey on condom use among 10–19 year-olds in Mpwapwa and Mbeya rural districts of Tanzania using a structured questionnaire. Associations between acceptability of condom promotion and distribution and each of the explanatory variables were tested using Chi Square. Multivariate logistic regression model was used to examine independent predictors of the acceptability of condom promotion and distribution using STATA (11) statistical software at 5% significance level.

**Results:**

Mean age of the 1,327 adolescent participants (50.5% being males) was 13.5 years (SD = 1.4). Acceptance of condom promotion and distribution was found among 37% (35% in Mpwapwa and 39% in Mbeya rural) of the adolescents. Being sexually active and aged 15–19 was the strongest predictor of the acceptability of condom promotion and distribution (OR = 7.78, 95% CI 4.65-12.99). Others were; not agreeing that a condom is effective in preventing transmissions of STIs including HIV (OR = 0.34, 95% CI 0.20-0.56), being a resident of Mbeya rural district (OR = 1.67, 95% CI 1.28-2.19), feeling comfortable being seen by parents/guardians holding/buying condoms (OR = 2.20, 95% CI 1.40-3.46) and living with a guardian (OR = 1.48, 95% CI 1.08-2.04).

**Conclusion:**

Acceptability of condom promotion and distribution among adolescents in Mpwapwa and Mbeya rural is low. Effect of sexual activity on the acceptability of condom promotion and distribution is age-dependent and was the strongest. Feeling comfortable being seen by parents/guardians buying or holding condoms, perceived ability of condoms to offer protection against HIV/AIDS infections, district of residence and living arrangements also offered significant predictive effect. Knowledge of these factors is vital in designing successful and sustainable condom promotion and distribution programs in Tanzania.

## Background

Successful condom promotion and distribution efforts require a better understanding of target populations’ mind-set of condoms before any intervention. Because of condoms’ scientifically known ability to protect humans against transmission and acquisition of human immuno-deficiency virus (HIV) and other sexually-transmitted infections (STIs) [1,2], their correct and consistent use is highly recommended [3]. In sub-Saharan Africa where condom use is very low [4], the challenge remains being how to maximize the use of condoms especially in areas with culturally rooted resistances to sexual behaviour change. Research attributes this to social values, perceptions, attitudes towards sex and condoms and traditional ways of practicing sex on one hand, and the systemic factors such as supply- and demand-related conditions in different social settings on the other [5-7]. While the need for promotion and distribution of condoms may be well-recognized by programs, understanding users’ contexts holds remarkable pertinence to the overall acceptability of condoms [8].

Despite condom promotion and distribution programs being several worldwide, extant evidence shows a weak evidence of increased condom use due to condom promotion and distribution [9]. This raises concerns regarding acceptability and uptake of condoms. To what extent do HIV/AIDS prevention programs fit into community-specific social structures in order to influence options and preferences regarding condoms? Analysts pinpoint a need for more systematic research that could help sexual health professionals among whom are those involved in designing interventions to use evidence in strategizing ways of increasing the cultural and individual acceptance of condoms and condom use [10]. Despite condoms being available – except recent report of inadequate coverage of female condoms [11] – there is vast evidence indicating persistence of incorrect and inconsistent use of condoms and escalating likelihood of condom discontinuation as duration of sexual relationship lengthens [12,13]. This ultimately impedes the success of HIV/AIDS prevention programs in the fight against AIDS and other STIs. Furthermore , analysts have recently noted an increasing attention among researchers on the assessment of consistency of condom use and non-compliance or errors associated with condom use [14,15].

Positive achievements in condom promotion efforts should also be acknowledged. For instance, in South Africa as in several African countries, national surveys continue showing progressive increases in rates of condom use at last sex [16]. Social marketing programs have attempted to promote condoms in Africa and many other low and high income countries by trying both subsidization and total pricing marketing approaches with remarkable success in enhancing the rate of condom use [17]. These efforts are commendable, even though evidence of condom use in the beginning of the relationship and later stoppage as the relationship deepens exist [16].

The HIV/AIDS pandemic in Tanzania as elsewhere in the world remains a disease of great public health importance and a challenge to socio-economic development that among other things lowers prospects for attaining the millennium development goals (MDGs). HIV/AIDS has devastating effects that are multifaceted, upshoting not only in health but also in the country’s economic growth, social welfare and all developmental structures [18]. About 1.2 million adult Tanzanians (age ≥15 years) were living with HIV in 2010 [19]. This was an appreciable decline since this number was 1.6 million in 2003 [20]. However, regional disparities in the HIV/AIDS severity still exist in Tanzania. For example, while in 2007 the overall national HIV/AIDS prevalence was 6%, it was as low as <2% in Arusha and as high as 16% in Iringa [20]. Records further show that in 2009, around 100,000 Tanzanians were newly infected with the HIV and 86,000 people died of AIDS in the same year [19]. Moreover, it is revealed that over 50% of new HIV/AIDS infections occur among young people aged between 15 and 24 years [21]. Extant evidence also indicate that adolescents are very much exposed to STIs, including HIV/AIDS due to their involvement in unprotected sex, multiple sexual partnerships and early sexual initiation [22,23]. Over 80% of transmissions and acquisitions of the AIDS virus in Tanzania and sub-Saharan Africa as a whole occur mainly heterosexually [24], which begins in early teens and pinnacles before age 30 years [18]. This calls for amplified prevention efforts that are sensitive among other things on population-specific social-cultural differences and needs.

Amongst the available ways of HIV prevention worldwide, the World Health Organization (WHO) acknowledges condoms as a key component of comprehensive HIV prevention [25]. Likewise, the government of Tanzania recognizes the role of condom use in preventing the spread of the HIV/AIDS and has thus been made an integral part of the warfare against the pandemic [26]. Initiatives such as Femina Health Information Project (Femina HIP) [27] and MEMA kwa Vijana Project [28] that aim at improving sexual and reproductive health services for young people exist locally. Even so, condom use among young people in Tanzania is exceptionally low, less than one-third of them reported condom use at their first sexual intercourse in 2007 [20] for example. Similarly, a study which was carried out recently in Tanzania among 10–19 year-olds found among other things that more than 60% of those who reported having had multiple sexual partners in the past year did not use condoms at the last sexual intercourse [29]. Further evidence of low and inconsistent condom use in Tanzania is available [30-32]. Overall, this suggests that condom promotion and distribution programs in the country have been less successful.

The current study is thus important because promotion and distribution of condoms alone may be inadequate if the degree of and what influences the acceptability of condoms is not well-understood. Condoms may be freely or cheaply available, but if people do not accept them or perceive their importance, no remarkable change in their consumption may be noticed. Therefore, it is important that factors that affect the acceptability of condom promotion and distribution are well understood in order to enhance quality and outcome of HIV-related programs and interventions. Apart from studies that have looked at condom use [22,29], our literature search revealed no study so far which has been conducted in Tanzania to assess the acceptability of condom promotion and distribution among 10–19 year-olds. This study thus responds to this knowledge gap in three objectives; (1) to quantify the proportion of 10–19 year-old adolescents that accept condom promotion and distribution among them and their peers, (2) for those that do not accept condom promotion and distribution, identify their reasons for not accepting condom promotion and distribution and (3) to assess factors associated with the acceptability of condom promotion and distribution among adolescents in Tanzania.

## Methods

### Study design and sampling

This study was cross-sectional in design. Data collection was conducted non-electronically using a standard questionnaire which was an interviewer-administered with many of its questions closed-ended. Field interviewers were adequately trained and after pilot survey, the questionnaire was finalized and the main survey study commenced shortly thereafter. The study areas were selected randomly using a multistage sampling technique which begun with identifying regions and later downsizing randomly to districts and within districts, selection of wards from which the primary schools and villages around were identified.

### Study area and study population

The study was conducted in two Tanzanian districts with different HIV prevalence namely, Mbeya rural (Mbeya region) and Mpwapwa (Dodoma region). Selection of these districts was justified by the evidence that the prevalence of HIV/AIDS in Tanzania varies with geographical localities (e.g. rural versus urban, roadside versus interior, etc.) [33]. Records show that Mbeya is one of the worst affected regions of Tanzania by the HIV/AIDS pandemic with prevalence of 14% [34,35]. In contrast, the HIV prevalence in Dodoma region was estimated at 5% in 2003–04 [35].

Unfortunately, there are scanty publications that give a full account of the socioeconomic characteristics of the two districts. However, national population and housing census and demographic and health survey (DHS) reports indicate that over 80% of the residents of both districts are engaged mainly in agriculture with small scale farm holdings where they use traditional un-mechanized technology. In Mpwapwa, livestock keeping is the second main economic activity of the majority. The district (Mpwapwa) is predominantly rural as majority of its residents live in rural areas. The reports show further that majority of people live in situations of poverty, albeit differences between rural and urban settings within and between the two districts. However, Mbeya rural seems to be in a relatively better position as reflected by other indicators such as proportions of people having access to formal education, health facilities, roads and household infrastructure [36,37].

Individual participants at school level were selected from a list of all eligible candidates at each school while those at a village level were identified from random households which were selected from a list of all households obtained from respective village leaders for each village. The participants were 10–19 year-olds.

### Variables and statistical analyses

A dependent or outcome variable for this study was acceptability of condom promotion and distribution among 10–19 year-old adolescents. Adolescents who reported that they agree with condom promotion and distribution among them and their peers were considered as being in acceptance while those who said that condoms should not be promoted and distributed among adolescents were considered as not being in acceptance with condom promotion and distribution. The variable was thus binary, with two categories as “accept” and “do not accept” condom promotion and distribution. The former (accept) was represented by a code “1” while the latter (not accept) was represented by a code “0”.

Independent (predictor/explanatory) variables included were sex, age, religion, district of residence and living arrangements. Other independent variables were sexual activity, perception of condoms’ protective effectiveness of STIs including HIV/AIDS, knowledge about places where condoms are available or distributed for free, experience with seeing or having heard about condoms, feelings about condom prices and how would one feel being seen by a parent or guardian holding or buying condoms.

The analysis began with describing the data in the form of one-way tabulations, bivariate analysis and later extended to multivariate analysis using logistic regression model. Since all the explanatory variables were categorical, the association between acceptability of condom promotion and distribution and each of the explanatory variables was tested using Pearson’s Chi Square. Selection of variables for inclusion in the multivariate model was based on the log-likelihood ratio test, whereby a variable was retained in the model if there was statistical evidence that its presence improved the model [38]. The model was finally checked for presence of interaction and adequacy before being approved as final. The whole process of data analysis was conducted using STATA (version 11) statistical software at 5% significance level.

### Ethics

An ethical approval for the parent study from which this paper stems was granted by the Medical Research Coordinating Committee (MRCC) of the National Institute for Medical Research (NIMR). Participation in the study was voluntary with an informed consent being signed by the respondent after which an interview followed. An assent for each of the participants under 18 years of age was given after receiving approval from their respective parents/guardians. The data were anonymous and their storage was careful and confidential.

## Results

### Background characteristics

Table 1 presents background characteristics of the 1,327 adolescent participants who were aged 13.5 years (SD = 1.4) on average. Sex composition was generally even at 50.5% and 49.5% of male and female adolescents respectively. Christianity constituted a majority (93.9%) of the participants and the rest were Muslims. In terms of district of residence, 45.6% were living in Mbeya rural district and the rest resided in Mpwapwa district. Nearly four-fifths (81.1%) reported that they live with their parents while the rest were living with guardians (people or relatives other than their parents).

**Table 1:** Background characteristics of the 10-19 year old participants in the analysis of acceptability of condom promotion and distribution in Mpwapwa and Mbeya rural districts of Tanzania, 2011 (N = 1, 327)

**Background characteristics**	**n**	**%**
**Sex**	**1, 322**	**100.0**
Male	668	50.5
Female	654	49.5
**Age (in years)**	**1,322**	**100.0**
10-14	1,061	80.0
15-19	266	20.0
Mean = 13.5, SD = 1.4	-	-
**Religion**	**1, 319**	**100.0**
Christian	1,239	93.9
Muslim	80	6.1
**District of residence**	**1, 327**	**100.0**
Mbeya rural	605	45.6
Mpwapwa	722	54.4
**Living arrangement**	**1, 311**	**100.0**
Lives with parent(s)	1, 063	81.1
Lives with guardian	248	18.9

SD = Standard Deviation, n = Number of respondents. Due to missing responses, some variable categories do not add up to 1, 327.

Results of the association between acceptability of condom promotion and distribution among 10–19 year-old adolescents and each of the explanatory variables are presented in Table 2. We found that overall, 36.9% of the 10–19 year-old adolescents were in acceptance of condom promotion and distribution among them and their peers as well. This proportion was 34.9% in Mpwapwa district and 39.7% in Mbeya rural district. The acceptability of condom promotion and distribution was associated with various background and non-background characteristics of the participants. Percentage distribution of the respondents that accept condom promotion and distribution in each category of the variables by district of residence are also presented in Table 2.

**Table 2:** Percentage distribution of 10-19 year-old adolescents that accept condom promotion and distribution among them and their peers by background and non background characteristics in Mpwapwa and Mbeya rural districts of Tanzania, 2011 (N = 1, 327)

		**Percent of adolescents that accept condom promotion and distribution**			
**Variable**	**Number of adolescents**	**Overall**	**District**		**P-value**
			**Mpwapwa**	**Mbeya rural**	
**Sex**	1,322	36.9	34.9	39.2	0.112
Male	668	38.9	37.8	40.3	
Female	654	34.7	32.1	38.1	
**Age (in years)**	1, 327	36.9	34.9	39.3	<0.001
10-14	1,061	33.0	28.3	37.8	
15-19	266	52.6	54.4	48.8	
**Religion**	1,319	36.9	34.9	39.2	0.284
Christian	1,239	37.2	35.5	39.2	
Muslim	80	31.3	28.8	38.1	
**Living arrangement**	1,311	37.0	35.0	39.4	0.037
Lives with parent(s)	1,063	35.7	34.8	36.7	
Lives with guardian	248	42.7	35.9	50.4	
**Sexual activity**	1,258	36.7	34.8	39.3	<0.001
Active	275	61.8	63.7	58.3	
Inactive	983	29.7	25.2	35.1	
**Ever heard or seen a condom**	1,325	37.0	34.9	39.5	0.263
No	97	42.3	20.0	44.8	
Yes	1,228	36.6	35.1	38.6	
**Agreeability of condom effectiveness in preventing transmission of STIs**	1,317	37.1	34.9	39.7	<0.001
Agree	1,070	39.6	38.4	41.1	
Do not agree	133	15.0	19.3	8.0	
Difficult to say	144	38.6	22.4	55.4	
**Do you know a place where condoms are available or distributed freely?**	1,323	36.9	35.0	39.2	0.011
No	1,072	35.3	34.3	36.3	
Yes	251	43.8	37.2	54.7	
**Feelings about condom price**	1,315	36.9	35.0	39.2	<0.001
Too costly	35	48.6	46.2	55.6	
Fair	392	47.2	44.2	55.1	
Don’t know	888	31.9	27.8	35.4	
**How would you feel being seen by a parent/guardian holding or buying a condom?**	1,298	37.1	34.9	39.8	<0.001
Comfortable	116	62.9	54.6	74.0	
Uncomfortable	1,182	34.5	32.9	36.6	

Due to missing responses, some variable categories do not add up to 1,327. P-values are based on Pearson’s Chi Square test between each of the variables and the overall acceptance of condom promotion and distribution.

By sex, the acceptability of condom promotion and distribution was 38.9% among male and 34.7% among female adolescents. The difference observed between them was not significant (P = 0.112). In terms of their age, 33.0% of the younger adolescents (10–14) reported acceptance of condom promotion and distribution. This proportion was 52.6% among older adolescents (15–19) and the difference was statistically significant (P < 0.001). Christian and Muslim adolescents were similarly accepting condom promotion and distribution (37.2% versus 31.3%) (P = 0.284). While the acceptability of condom promotion and distribution was 37.5% among adolescents living with their parents, it was 42.7% among those living with guardians and the difference observed was statistically significant (P = 0.037).

With respect to sexual activity, it was found that sexually active adolescents were nearly two times as many sexually inactive ones to accept condom promotion and distribution (61.8% versus 29.7%) and the difference was statistically significant (P < 0.001). However, despite the fact that a majority of the adolescents (92.7%) had had seen or heard of a condom, their level of acceptance of condom promotion and distribution was not different from that observed among adolescents who had never heard or seen a condom (P = 0.263).

The acceptability of condom promotion and distribution was also examined across levels at which the respondents were in agreement with the claim that condoms are effective in preventing transmission of STIs including HIV if appropriately used. We found that 39.6% of those who were in agreement with the claim were also in acceptance with condom promotion and distribution. This proportion was 15.0% and 38.6% among those who reported that they do not agree and those who said that it is difficult to say respectively regarding the claim on condom effectiveness. These differences were statistically significant (P < 0.001). Likewise, the proportion of adolescents that reported that they know places where condoms are available or distributed for free exceeded that for adolescents who did not know such places (43.8% versus 35.3%) and the difference was significant (P = 0.011). Regarding perceptions on condoms prices, 48.6% of those who felt that condoms are too costly reported that they accept condom promotion and distribution. This proportion was 47.2% and 31.9% among those who reported that the price is fair and those who reported that they do not know the prices respectively, and the difference observed was statistically significant (P < 0.001).

On the other hand, 62.9% of the adolescents who said that they would feel comfortable being seen by a parent or guardian holding or buying condoms reported to find the issue of condom promotion and distribution acceptable to them. The corresponding proportion among those who said that they would feel uncomfortable if they are seen by a parent or guardian holding or buying condoms was 34.5% and the difference between them was statistically significant (P < 0.001).

### Multivariate logistic regression results

Adjusted factors associated with acceptability of condoms promotion and distribution among 10–19 year-old adolescents in Mpwapwa and Mbeya rural districts of Tanzania is presented in Table 3. The findings reveal presence of an interaction between sexual activity and age in the prediction of acceptability of condom promotion and distribution. The effect of sexual activity on the acceptability of condom promotion and distribution is age-dependent and vice versa. Adolescents who were sexually active and aged 15–19 years were almost 8 times more likely to accept condom promotion and distribution compared to those who were sexually inactive and aged 10–14 (OR = 7.78, 95% CI 4.65-12.99). Similarly, acceptance of condom promotion and distribution was nearly 6 times more likely among adolescents who were sexually active compared to sexually inactive ones and aged 15–19 (OR = 5.91, 95% CI 3.24-10.77). It was also twice more likely that the sexually active group will accept condom promotion and distribution compared to their sexually inactive counterpart and aged 10–14 (OR = 2.34, 95% CI 1.61-3.40). Likewise, compared to 10–14 year-olds, adolescents in the age group 15–19 and sexually active were around 3 times more likely to accept condom promotion and distribution (OR = 3.32, 95% CI 1.86-5.92).

**Table 3:** Multivarate logistic regression model of correlates of acceptability of condom promotion and distribution among 10-19 year-old adolescents in Mpwapwa and Mbeya rural districts of Tanzania, 2011 (n = 1,191)

**Covariate**	**Odds Ratio (OR)**	**95% Confidence Interval (CI)**
**Sexual activity X Age (in years)**		
Active and age 15-19 versus inactive and age 10-14	7.78***	4.65-12.99
Active versus inactive, age 15-19	5.91***	3.24-10.77
Active versus inactive, age 10-14	2.34***	1.61-3.40
Age 15-19 versus age 10-14, active	3.32***	1.86-5.92
Age 15-19 versus age 10-14, inactive	1.32	0.89-1.95
**Agreeability of condom effectiveness in preventing transmission of STIs**		
Agree (ref.)	1.00	-
Do not agree	0.34**	0.20-0.56
Difficult to say	1.18	0.74-1.87
**District of residence**		
Mpwapwa (ref.)	1.00	-
Mbeya rural	1.67***	1.28-2.19
**How would you feel being seen by a parent/guardian holding or buying a condom?**		
Uncomfortable (ref.)	1.00	-
Comfortable	2.20***	1.40-3.46
**Living arrangement**		
Lives with parent(s) (ref.)	1.00	-
Lives with a guardian	1.48**	1.08-2.04
**Sex**		
Female (ref.)	1.00	-
Male	0.77	0.59-1.01
**Feelings about condom price**		
Too costly (ref.)	1.00	-
Fair	0.73	0.33-1.62
Don’t know	0.48	0.22-1.05
**Do you know a place where condoms are available or distributed freely**		
No (ref.)	1.00	-
Yes	1.04	0.74-1.44

***P < 0.001, **P < .05; ref. = reference/baseline category; Variables are adjusted for one another and the Hosmer-Lemeshow goodness-of-fit test showed that the model fits the data adequately (P = 0.420).

Furthermore, adolescents who reported that they perceive condoms as being ineffective in preventing transmission of STIs including HIV were 66% less likely than those who did perceive so to accept condom promotion and distribution (OR = 0.34, 95% CI 0.20-0.56). On the other hand, those who were residents of Mbeya rural district were 67% more likely than residents of Mpwapwa district to accept promotion and distribution of condoms among them (OR = 1.67, 95% CI 1.28-2.19).

Another important factor was living arrangement. Adolescents who reported that they live with guardians were 48% more likely than those who reported that they live with their own parents to accept condom promotion and distribution (OR = 1.48, 95% CI: 1.08-2.04). Similarly, adolescents who reported that they would feel comfortable being seen by a parent or guardian holding or buying condoms were almost twice as likely as those who would feel uncomfortable under such scenarios to accept condom promotion and distribution among them and their peers (OR = 2.20, 95% CI 1.40-3.46).

Finally, there were no evidence of predictive effect of condom promotion and distribution due to sex (male versus female: OR = 0.77, 95% CI 0.59-1.01), feelings about condom price (fair versus too costly: OR = 0.73, 95% CI 0.33-1.62; don’t know versus too costly: OR = 0.48, 95% CI 0.22-1.05) and knowledge of places where condoms are available or distributed for free (OR = 1.04, 95% CI 0.74-1.44).

### Reasons for not accepting condom promotion and distribution

**Figure 1:**
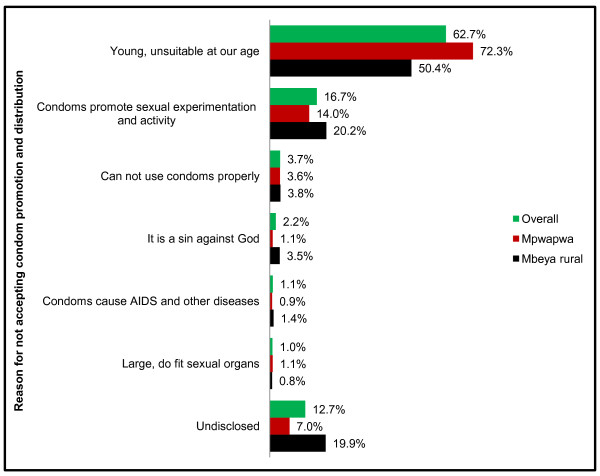
Reasons reported by 10–19 year-old adolescents (n = 837) for not accepting promotion and distribution of condoms among them and their peers in Mpwapwa and Mbeya rural districts of Tanzania, 2011.

Study participants who reported that there should be no promotion and distribution of condoms among them and their peers (n = 837), subsequently reported reasons for their stance. Distribution of the reasons reported (percent of adolescents that reported each reason) by district of residence are presented in Figure 1.

A majority (62.7%) of the adolescents reported that they are too young to be given condoms and condom promotions. This proportion was 72.3% and 50.4% in Mpwapwa and Mbeya rural districts respectively. The participants argued that condoms are suitable for adults, not children of their age because they should not be engaging in sexual activities before marriage. Reports from 16.7% of the respondents further indicated that condoms should not be promoted and distributed to adolescents because doing so would influence not only the sexually active ones to engage in heterosexual affairs, but also those who have never had sex would want to experiment the use of condoms, an act which would mostly lead into an occurrence of a sexual intercourse (early sexual initiation), thus expose them to the risk of STIs. A few (3.7%) of the respondents also reported that there should be no condom promotion and distribution among adolescents because they do not know how to use such products properly. A religiously-related reason was also reported by 2.2% of the adolescents that having a premarital sex is sinning or committing an abomination before God. Though few, a perception that condoms cause AIDS and other diseases emerged among 1.1% respondents and also that condoms are large and do not fit their sexual organs (1.0%). Thirteen percent (12.7%) of the respondents did not disclose any reason for rejecting condom promotion and distribution.

## Discussion

This study sought to assess the extent of and factors associated with acceptability of condom promotion and distribution among 10–19 year-old adolescents in Mpwapwa and Mbeya rural districts of Tanzania, using cross-sectional data collected in these districts in 2011. Adolescent participants who reported that there should be no condom promotion and distribution among them were subsequently asked why they think so. The findings reveal a small proportion (37%) of adolescents that agree that promotion and distribution of condoms among them and their peers both in- or out- of schools is worthwhile. Unfortunately, we found no comparable literature apart from studies that report prevalence of condom use at the last sexual intercourse among 10–19 year-olds in some districts of Tanzania [22,29]. While this may partly represent the extent of acceptance of promotion and distribution of condoms, it does not account for those who are in acceptance of condom promotion and distribution but are not sexually active.

Participants who reported that they do not agree (rejection) with condom promotion and distribution among them and their peers reported that they are too young to be sensitized about condoms and that promotion and distribution of condoms among them would encourage sexual practices as they explore or experiment the use of condoms. This suggested among other things that promotion and distribution of condoms among adolescents would promote sexual initiation. Although the perception that condom promotion among adolescents might increase sexual activity has been reported [39,40], other studies have shown that the two are not related [41,42]. Further research is thus required to provide clarifications.

Around a quarter of the adolescents reported that they were sexually active and the multivariate model revealed that being sexually active and aged 15–19 was a strongest predictor of the acceptability of condom promotion and distribution. It is likely that perceived risk of STIs including HIV/AIDS and unintended pregnancies (especially females) which may dictate one’s need for protection, together with the possibility of having had a longer duration of exposure to promotions or advertisements of condoms and HIV/AIDS sensitization messages may altogether have influenced positively the acceptability of condom promotion and distribution among older and sexually active adolescents. Other studies have found that older adolescents are more likely than younger ones to use condoms [29], implying a varied degree of acceptability of either the exposure to, or a positive perception on roles that condoms play.

Empirical and anecdotal evidences show considerable attention being on the importance of geographical factors including location on the distribution, accessibility and eventually acceptability and utilization of health services [43]. The present study shows that the respondents from the two districts were of a different degree in the acceptability of condom promotion and distribution. It was noted that adolescent residents of Mbeya rural district were more likely than their counterparts in Mpwapwa district to accept condom promotion and distribution. While this may have implications on the actual use of condoms, it may be an indicator of the extent to which the HIV/AIDS prevention programs have penetrated the areas differently. Since Mbeya is one of the regions in Tanzania with highest HIV/AIDS prevalence, considerable HIV prevention efforts that have gone into such areas [35] may have positively shaped people’s behaviour and outlook of condoms thus more likely that the acceptance of condom promotion and distribution in such places is thus higher than elsewhere. Considering that the use of condoms partly result from favourable acceptance of their distribution, the spatial disparities observed may also be linked to differences in access to information and communication as similarly observed elsewhere [33,44]. The differences in cultures, values and norms that are partly linked to geographical locations also count remarkably [44]. Further research is however required to elucidate how best HIV/AIDS prevention programs can be integrated into existing socio-cultural structures using (where possible) local infrastructures as a way of increasing their acceptability and sustainability.

Family ties also seem to be important in explaining the observed difference in the acceptability of the promotion and distribution of condoms among adolescents. For instance, the present study reveal that adolescents who reported that they would feel comfortable if they were seen by a parent or guardian buying or holding condoms were more likely than those who said that they would feel uncomfortable to accept condom promotion and distribution. Moreover, those who were living with their guardians were more likely than those living with their own parents to accept condom promotion and distribution. It may be that adolescents who live with guardian(s) experience a higher level of autonomy when it comes to making decisions and choices on their sexually-related matters than those living with their parents. Exavery et al. [29] in their recent study of relationship between multiple sexual partnerships and condom use report on socio-cultural illegality of sexual practices among young unmarried adolescents. The study points out that an occurrence of a sexual intercourse among unmarried adolescents is a secret action in attempt to avoid a portrayal of behavioural disobedience or misconduct to parents, relatives and the community at large and may be associated with the belief that condom promotion and distribution to adolescents is a sinful attempt.

Parents or guardians and the community at large have key roles to play in shaping the ultimate behaviours of children. Even as parents are advised to be clear about their own values, attitudes and talking to their children early and establish rules and expectations of behaviour, they should strive to be role models of their children. It is recognized that parents’ behaviours have consequential impacts in the overall behavioural outcomes of their children [45], thus important that they behave well for the benefit of children.

Furthermore, acceptance of condom promotion and distribution was less likely to be noted among those who were not in agreement with the claim that a condom is effective in preventing transmission of STIs including HIV/AIDS than those who were in agreement. This indicates how the value of condoms in the fight against STIs may be downgraded due to individual or social misconceptions and stigma about condoms. This highlights the need for condom promotion and distribution programs and interventions to clarify myths and clearly explain condom effectiveness and emphasize correct and consistent use of it at each sexual encounter for better sexual and reproductive health outcomes of users.

### Limitations

The data collected during the fieldwork was self-reported, therefore being difficult to verify and may have been affected by the respondents’ recall bias. The present findings may not be generalized to represent the whole country since only two districts were surveyed. In addition, since the study was cross-sectional in design, no causal inferences may be drawn from our findings because the dependent variable - acceptability of condom promotion and distribution - and the independent variables were all measured at the same time thus difficult to establish temporality.

## Conclusions

The proportion of 10–19 year-old adolescents that accept condom promotion and distribution in Mpwapwa and Mbeya rural districts of Tanzania is generally low. Factors such as sexual activity, age, sex, living arrangements, place of residence, confidence when buying or accessing condoms and perceptions about condom effectiveness require important consideration in designing workable and sustainable HIV/AIDS control programs that advocate condom promotion and distribution among adolescents.

Therefore, efforts aimed at changing negative perceptions about condoms on religious and social morality grounds as well as in relation to the protective efficacy of condoms are crucial among Tanzanian adolescents. This can be achieved through a rigorous context-specific and social-cultural-based sexual and reproductive health education, and should be prioritized as an integral component for successful HIV/AIDS prevention programs in Tanzania.

## Competing interests

The authors declare that they have no competing interests.

## Authors’ contributions

AE conceptualized the research question, participated in designing the study and played a major role on the statistical analyses with close assistance of GMM and JR. AE and JR also drafted the manuscript. GMM conceived the parent study, lead the design of it and established the first draft of the full proposal of the parent study, wrote the first and final drafts of the technical research report and reviewed the first and final draft versions of the present manuscript. AKM and JJM assisted greatly GMM in the conception and proposal writing for the parent study. All the rest co-authors critically reviewed the manuscript and approved the final draft. MNM was also an overseer of the administrative and other logistical aspects of the study among a series of related studies. All authors read and approved the final manuscript.

## Pre-publication history

The pre-publication history for this paper can be accessed here:

http://www.biomedcentral.com/1471-2458/12/569/prepub
